# Efficacy and safety of metformin in the management of type 2 diabetes mellitus in older adults: a systematic review for the development of recommendations to reduce potentially inappropriate prescribing

**DOI:** 10.1186/s12877-017-0574-5

**Published:** 2017-10-16

**Authors:** Lisa Schlender, Yolanda V. Martinez, Charles Adeniji, David Reeves, Barbara Faller, Christina Sommerauer, Thekraiat Al Qur’an, Adrine Woodham, Ilkka Kunnamo, Andreas Sönnichsen, Anna Renom-Guiteras

**Affiliations:** 1 Institut für Allgemeinmedizin und Familienmedizin, UWH, Witten, Germany; 2 NIHR School for Primary Care Research, University of Manchester, Manchester, UK; 3 Faculty of Medicine, Jordan University of Science and Technology, Irbid, Jordan; 4 Duodecim Medical Publications Ltd, Helsinki, Finland; 5 Department of Geriatrics, University Hospital Parc de Salut Mar, Barcelona, Spain

**Keywords:** Systematic review, Metformin, Type 2 diabetes mellitus, Inappropriate prescribing, Older people, Elder, Elderly

## Abstract

**Background:**

Metformin is usually prescribed as first line therapy for type 2 diabetes mellitus (DM2). However, the benefits and risks of metformin may be different for older people. This systematic review examined the available evidence on the safety and efficacy of metformin in the management of DM2 in older adults. The findings were used to develop recommendations for the electronic decision support tool of the European project PRIMA-eDS.

**Methods:**

The systematic review followed a staged approach, initially searching for systematic reviews and meta-analyses first, and then individual studies when prior searches were inconclusive. The target population was older people (≥65 years old) with DM2. Studies were included if they reported safety or efficacy outcomes with metformin (alone or in combination) for the management of DM2 compared to placebo, usual or no treatment, or other antidiabetics. Using the evidence identified, recommendations were developed using GRADE methodology.

**Results:**

Fifteen studies were included (4 intervention and 11 observational studies). In ten studies at least 80% of participants were 65 years or older and 5 studies reported subgroup analyses by age. Comorbidities were reported by 9 studies, cognitive status was reported by 4 studies and functional status by 1 study. In general, metformin showed similar or better safety and efficacy than other specific or non-specific active treatments. However, these findings were mainly based on retrospective observational studies. Four recommendations were developed suggesting to discontinue the use of metformin for the management of DM2 in older adults with risk factors such as age > 80, gastrointestinal complaints during the last year and/or GFR ≤60 ml/min.

**Conclusions:**

On the evidence available, the safety and efficacy profiles of metformin appear to be better, and certainly no worse, than other treatments for the management of DM2 in older adults. However, the quality and quantity of the evidence is low, with scarce data on adverse events such as gastrointestinal complaints or renal failure. Further studies are needed to more reliably assess the benefits and risks of metformin in very old (>80), cognitively and functionally impaired older people.

**Electronic supplementary material:**

The online version of this article (doi:10.1186/s12877-017-0574-5) contains supplementary material, which is available to authorized users.

## Background

Type 2 diabetes mellitus (DM2) is a prevalent chronic disease worldwide. Around 9% of adults have DM2, increasing to more than 20% of those aged 65 years or older [1, 2]. DM2 and its complications are an important cause of morbidity, and people with DM2 have substantially reduced life expectancy [3]. Duration of DM2 and degree of metabolic control are important factors determining the prognosis for people with DM2 [4]. However, the use of drugs for managing DM2 has been associated with preventable drug-related causes of admission to emergency units in older populations [5–7].

Metformin is one of the most widely prescribed first and second line oral glucose-lowering drugs. While it has low risk for hypoglycaemia, the risk for gastrointestinal effects is higher and it is contraindicated in patients with renal insufficiency [8–10]. Renal function declines with age and, therefore, should be monitored closely in older adults who are prescribed metformin [11, 12]. Clinical guidelines (in the United Kingdom, Canada and Australia) have advised that the use of metformin is contraindicated, or that lower doses be used, depending on renal function [13]. The use of metformin has also been associated with a higher risk of lactic acidosis but this has not been widely reported [14].

Currently, there is little empirical data about patient safety and effectiveness data on to the use of oral antidiabetics including metformin among older adults. Evidence-based clinical guidelines for the treatment of DM2 have acknowledged the lack of direct evidence in older people [10]. STOPP/START criteria version 2 considered metformin as a potentially inappropriate medication for older people with severe renal failure [15]. Inappropriate prescribing may involve the prescription of a wrong dose, the lack of a clear indication or the lack of evidence-base, among others [16].

The objectives of this systematic review (SR) are:

- to identify and collect existing literature on the risks and benefits of use of metformin in the treatment of DM2 in older adults,
- to assess the quality of the evidence identified, and develop recommendations when to discontinue or to adjust the dose of metformin in the treatment of DM2 in older adults.

This evidence was used to develop recommendations on discontinuation or dose adjustment of metformin in older people for the management of DM2 in order to reduce potentially inappropriate prescribing. These recommendations will be used in the electronic decision support tool of the “Polypharmacy in chronic diseases: Reduction of Inappropriate Medication and Adverse drug events in elderly populations by electronic Decision Support” (PRIMA-eDS) project [16].

## Methods

This systematic review was developed following the methods proposed by both the Cochrane Handbook for Systematic Reviews of Interventions [17] and the Preferred Reporting Items for Systematic Reviews and Meta-Analyses (PRISMA) [18]. A full description of the methods has been published previously [19].

### Study inclusion criteria

#### Types of studies

We included systematic reviews, meta-analyses, controlled interventional studies and observational studies reporting on risks and benefits of the use of metformin in the treatment of DM2 in older adults. We excluded abstracts, pooled analyses, editorials, opinion papers, case reports, case series, narrative reviews, letters, and qualitative studies.

#### Types of participants

The population of interest were older people with DM2. We considered the age of 65 as cut-off point for defining older people, which has been traditionally used because of its association with retirement age in some developed countries [20–22]. The criteria for inclusion in this systematic review were:

For existing systematic reviews and meta-analyses:

- overall mean or median age ≥ 65 years; or
- overall mean or median age < 65 with subgroup analysis reporting on participants ≥65 years; or
- overall mean or median age not reported but 80% or more of the included studies reporting a mean or median age ≥ 65 years.

For individual controlled interventional studies and observational studies:

- ≥80% of participants ≥65 years; or
- <80% of participants ≥65 years with subgroup analysis reporting on participants ≥65 years.

#### Types of interventions

Studies reporting on the efficacy and/or safety of metformin for the management of DM2 were included irrespective of whether metformin was prescribed as monotherapy or in combination with any other. Included studies compared metformin versus placebo, usual or no treatment, and other drugs to treat DM2 or a non-pharmacological intervention.

#### Types of outcomes

The following clinically relevant endpoints were included either as primary or secondary outcomes:

- Quality of life
- Mortality
- Life expectancy
- Hospitalisations
- Cognitive impairment or cognitive status
- Functional impairment or status
- Cardiovascular event including stroke
- Renal failure
- Composite end points including any of the above (extraction of individual outcomes was undertaken if reported by original studies)
- Adverse drug event including hypoglycaemia
- Any of the above evaluated as safety endpoints.

Studies evaluating only glycaemic control or lactate levels. To aid interpretation of findings outcomes were classified into two tiers according to their anticipated impact on longer-term health and quality of life: Tier 1 outcomes have shorter-term impact including hypoglycaemia and adverse events (including serious adverse events); tier 2 outcomes have longer-term impact including, but not limited to, cardiovascular and cerebrovascular events, related admissions, and death.

#### Setting

All settings were included.

#### Language

Language restrictions were not applied for study searches. However, the inclusion of studies was restricted to languages that could be read by the research team English, German, Finish, Italian, and Spanish.

### Search method

Database searches were conducted by YVM and AW following staged methodology comprising four sequential literature searches. Each search being performed only if the preceding one yielded high quality results or if evidence insufficient to enable any evidence based recommendations to be made. Each search was conducted on 09 December 2015 using the OVID interface for each database. The searches included the following databases and types of studies:

- Search 1: Systematic reviews and meta-analyses in the Cochrane database of Systematic Reviews (2005 to 2015) and the Database of Abstracts or Reviews of Effects (1991 to 2015).
- Search 2: Systematic reviews and meta-analyses in MEDLINE and MEDLINE (R) In-Process & Other Non-Indexed Citations (1946 to 2015), EMBASE (1974 to 2015), Health Technology Assessment (HTA) (2001 to 2015) and International Pharmaceutical Abstracts (IPA) (1970 to 2015).
- Search 3A: Interventional and observational studies meeting eligibility criteria included in systematic reviews which did not meet the inclusion criteria for searches 1 and 2
- Search 3B: Additional controlled interventional and observational studies identified from MEDLINE, MEDLINE (R) In-Process & Other Non-Indexed Citations, EMBASE, HTA and IPA published since 2011.

References of included studies were checked to identify further articles for inclusion, and we also considered studies identified from manual searches and snowballing. Protocols for yet-to-be published studies were collected to inform future updates of this systematic review. Studies excluded after full-text check are listed in Additional file 1 together with reasons for exclusion.

The PICOS-framework was used to develop the search terms (population: older people, intervention: metformin, comparison: no limits, outcomes: see list above “Types of outcomes” and study design: systematic reviews, meta-analyses, controlled interventional studies and observational studies). We also developed search filters specific for different study designs, described in detail in the protocol [19]. Additional file 2 lists the full search terms for each search (i.e. search 1, 2 and 3B).

### Data management

Search results were uploaded to Endnote X7 reference management software where search results were retrieved and de-duplicated.

### Selection of studies

Two reviewers assessed titles and abstracts from each search independently to identify studies to consider for inclusion. Full manuscripts were then obtained for all titles and abstracts meeting the inclusion criteria or where there was any uncertainty about inclusion. YVM, ARG, CA, BF, CS and LS were involved in this task.

Reviewers discussed any disagreement about studies to include. AS was consulted when YVM and ARG could not reach an agreement on whether or not to include a study. YVM and ARG were consulted when CA, CS, BF and LS could not reach agreement.

### Data extraction

YVM, ARG, CA, BF, CS and LS (reviewers) independently conducted data extraction of the included studies using a standardised and piloted data collection form previously published with the protocol [19]. This extraction form included information related to the study design and aim, characteristics of participants (i.e. age, sex, setting, comorbidity, use of concomitant medications, functional status, and cognitive status), the intervention (i.e. metformin) and comparison, time to follow-up, and reported outcomes. Completeness and accuracy of data extraction was then double-checked by a second reviewer.

### Quality appraisal

For each study design we used separate validated assessment tools to evaluate quality (AMSTAR) [23, 24] was used for systematic reviews/meta-analyses, for intervention studies the Cochrane Collaboration’s tool for assessing risk of bias [17] was used, and for observational studies the Critical Appraisal Skills Programme (CASP) [25, 26].

### Dealing with duplicate and companion publications

All relevant data from publications relating to a single primary study were included. The staged approach carries a risk of ‘double counting patients whose trials are included in a systematic review. Any such instances have been identified, reported and corrected for in our data synthesis.

### Data synthesis

A narrative synthesis describing all included systematic reviews, meta-analyses, intervention and observational studies, participants and findings was carried out. The included studies were highly heterogeneous in comparison treatments, length of follow-up, type of design, and definition of outcomes; therefore no additional meta-analyses were performed. The quality of the included studies is also reported.

### Identification of “references of interest” for the development of recommendations

During the search process, reviewers identified additional references which did not fulfil the inclusion criteria of the SRs but which they considered of interest for the development of recommendations according to the methodology described by Martinez-Renom Guiteras (2016) [19].

### Development of recommendations

Included studies and references of interest were summarised in a document used by the research team to develop and discuss recommendations to discontinue the use of metformin for the management of DM2 in older people including: a) study design or type of reference, target population and sample size, metformin dose (if available) and comparison groups, outcomes, main results, subgroup analysis if applicable; b) quality appraisal ratings of included studies; and c) proposed recommendations. Each recommendation was given a strength (weak or strong) and quality (low, moderate or high) rating following the Grading of Recommendations Assessment, Development and Evaluation (GRADE) methodology [27–29]. Recommendations were written following a standardised schema and reflecting the strength and the quality of the evidence. The Finnish team of editors from Duodecim Medical Publications Ltd. participated in the later stages and approved the recommendations.

## Results

### Results of the search

Searches 1, 2, 3A and 3B were all conducted. Search 1 identified one relevant meta-analysis, by Lamanna et al. [30] which did not provide summary results for our SR targeted at old age and was excluded. However eligible individual studies were identified from it and added to search 3A. The date of the search by Lamanna (2011) [30] was used as the start date for our search for additional individual studies under search 3B. No relevant meta-analyses were identified from search 2.

**Fig. 1:**
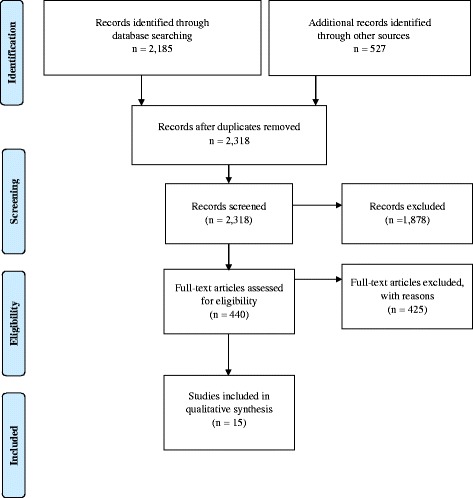
Preferred Reporting Items for Systematic Reviews and Meta-Analyses (PRISMA) flow diagram

In total 2185 records were found through initial database searching (126 from search 1, 175 from search 2, 1884 from search 3B). Additionally, we identified 66 records from search 3A (individual studies from excluded systematic reviews and meta-analyses) and 461 records from reference lists of included studies. After removing duplicates, we screened 2318 records and excluded 1878 checking titles and abstracts. We assessed 440 full-texts for eligibility and excluded 425. Main reasons for exclusion were wrong population, wrong intervention and wrong outcome. We included 15 studies reported by one publication each. The PRISMA flow diagram is presented in Fig. 1.

### Included studies

Fifteen studies were included [31–45] including 426,549 participants of all ages of which 230,229 were 65 years and older. However, analyses of efficacy and risks of antidiabetic drugs did not always include all participants. Table 1 shows the summary of the study characteristics of included studies.

**Table 1:** Summary of study characteristics

Authors and publication year	Type of study	Aim	Sample size and information about the amount of older participants^a^	Follow-up	Outcomes and measurement tools if applicable
Cryer 2005 [31]	Randomised, open label, parallel-group, multicentre, clinical trial	To evaluate the risk of lactic acidosis or other SAEs with metformin, under usual care conditions.	P: 8732 *P* ≥ 65 years: 3084 P using metformin (metformin only or in combination with SU or non-SU oral agent): 2515 P under usual care (SU, TZD, insulin, or any other non-metformin monotherapy or combination therapy): 569	12 months	Incidence of SAEs^c^, hospitalization, and death.
Evans 2010 [32]	Population-based prospective cohort study	To examine the efficacy of metformin and SU in patients with DM2 and CHF.	P: 422 *P* ≥ 65 years: 365 P using SU: 191 P using any metformin (alone/combination): 174	1 year and at end of follow-up (death, loss to follow-up, or end of 10 year study window)	All-cause mortality
Hung 2013 [33]	Population-based retrospective cohort study	To assess the risk of non-fatal cardiovascular events among patients with DM2 who are taking metformin monotherapy, glimepiride or glyburide.	P: 1159 *P* ≥ 71 years: 231 P using glyburide: 72 P using glimepiride: 50 P using metformin: 109	3 months	Incidence of non-fatal cardiovascular events including coronary artery disease, peripheral artery disease, stroke and heart failure.
Inzucchi 2005 [34]	Retrospective cohort study	To determine the impact of insulin sensitizers on outcomes in diabetic patients after hospitalization with AMI.	P: 8872 *P* ≥ 65 years: 8872 P using no insulin sensitizer: 6641 P using metformin: 1273 P using TZD: 819 P using metformin + TZD: 139	1 year	Time from hospital discharge to death from any cause censored at 1 year of discharge. Time to first readmission for MI, first readmission for heart failure, and first readmission for any cause up to 1 year after discharge.
Janka 2007 [35]	Parallel-group, open-label, randomized, multinational clinical trial	To investigate whether the safety and efficacy benefits of initiating insulin therapy with glargine and continued OADs, versus switching to premixed insulin, as previously reported, were also observed in the subset of patients aged 65 and older.	P: 130 *P* ≥ 65 years: 130 P using insulin glargine + glimepiride and metformin: 67 P using premixed insulin: 63	24 weeks	Hypoglycaemic events and their frequency.
Josephkutty et al. 1990 [36]	Randomised double-blind cross-over study	Efficacy, metabolic effects, and acceptability of metformin were compared with tolbutamide in 20 diabetic patients aged between 65 and 95 years.	P: 20 *P* ≥ 65 years: 20 P using metformin/tolbutamide: 10 P using tolbutamide/metformin: 10	3 months with each treatment	Side-effects of drug treatment.
Lapane et al. 2015 [37]	Retrospective cohort study	To evaluate the extent to which SU use was associated with fractures and falls among nursing home residents with DM2.	P: 11,958 *P* ≥ 65 years: 10,916 P using SU: 5128 P using biguanide: 5788	Median 683 days (range: 1–1002 days)	Severe hypoglycaemia, falls, and fractures occurring in parts of the body typically associated with falling.
MacDonald 2010 [38]	Case-control study	To examine outcomes in patients with DM2 and heart failure and to determine whether outcomes were associated with antidiabetic drug therapy.	P: 3266 *P* ≥ 65 years: 3102 Cases (P with DM2 and heart failure who died): 1633 Controls (P with DM2 and CHF alive): 1633	Average 11 years	All-cause mortality.
Masoudi 2005 [39]	Retrospective cohort study	To assess the relationship between the prescription of insulin-sensitizing agents (metformin and/or TZD) and death or readmission ofelderly diabetic patients initially admitted with heart failure in a cohort derived from the National Heart Care Project (NHC).	P: 5296 *P* ≥ 65 years: 5296 P using metformin: 1861 P using TZD: 2226 P using no insulin sensitizer: 12,069	1 year	Time from hospital discharge to death due to any cause, time to first readmission for any cause or for heart failure, proportions of patients who died or were readmitted at least once in the year after discharge, rates of readmission for the primary diagnosis of metabolic acidosis.
Moore et al. 2013 [40]	Cross sectional study	To investigate the associations of metformin, serum vitamin B12, calcium supplements, and cognitive impairment in patients with DM2.	P: 1354 *P* ≥ 65 years: 1164 P with DM2/impaired glucose tolerance: 126 P using metformin: 35 P no using metformin: 91	Not applicable	Cognitive performance measured with the Mini-Mental State Examination (MMSE).
Roumie 2012 [41]	Retrospective cohort study	To compare the effects of SU and metformin monotherapy on CVD outcomes (AMI and stroke) or death.	P: 253,640 *P* ≥ 65 years: 118,014 P using metformin: 64,009 P using SU: 54,005	12 months	Hospitalization for AMI or stroke, or death. Composite of AMI and stroke events only.
Roussel et al. 2010 [42]	Prospective, observational registry	To assess whether metformin use was associated with difference in mortality after adjustment for baseline differences and for the propensity to receive metformin among diabetics with established coronary artery disease, cerebrovascular disease, or peripheral arterial disease.	P: 19,553 *P* ≥ 65 years: 12,649 P using metformin: 4389 P without metformin: 8260	2 years	2-year all-cause mortality. Cardiovascular death and first-occurring event among death, MI, or stroke.
Schweizer 2009 et al. [43]	Randomized, active-controlled, parallel-group study	To compare the efficacy and tolerability of vildagliptin with metformin in elderly patients with DM2.	P: 335 *P* ≥ 65 years: 335 P using metformin: 166 P using vildagliptin: 169	24 weeks	AEs.
Solomon 2009 [44]	Retrospective cohort study	To determine the association between TZD use and fracture risk among older adults with DM2.	P: 20,291 *P* ≥ 65 years: 20,291 P using TZD: 2347 P using SU: 13,709 P using metformin: 4235	Follow-up ended at the first of any of the following events: death, loss of eligibility for Medicare or the drug benefit program, 180 days after the last dosage of oral hypoglycaemic agent, or end of follow-up.	Incidence of fracture within the cohort.
Tzoulaki 2009 [45]	Retrospective cohort study	To investigate the risk of incident MI, congestive heart failure, and all-cause mortality associated with prescription of OADs monotherapies and combinations.	P: 91,521 *P* ≥ 65 years: 45,760 P using FGS: 4764^b^ P using SGS: 40,266 ^b^ P using rosiglitazone: 4437^b^ P using rosiglitazone combination: 4642^b^ P using pioglitazone (alone/combination): 1879^b^ P using other drugs or combinations: 21,994^b^ P using metformin: 37,357^b^	Mean follow-up 7.1 years	First occurrence of incident MI, CHF, and all-cause mortality.

*SAEs* serious adverse events; *P* participants; *SU* sulfonylurea; *AEs*: adverse events; *OADs* oral antidiabetic; *CHF* chronic heart failure; *DM2* type 2 diabetes mellitus; *TZD* thiazolidinedione; *AMI* acute myocardial infarction; *CVD* cardiovascular disease; *MI* myocardial infarction

^a^unreported counts were derived from available data where possible

^b^The sum of patients in each subgroup is greater than the number of participating patients, as the study design allowed patients to take different drugs at different time periods

^c^SAEs comprised any experience that was fatal, life-threatening, permanently or substantially disabling, resulted in permanent or significant disability or incapacity, required or prolonged hospitalization, an important medical event that jeopardized the patient or required intervention to prevent a serious outcome, a congenital abnormality, a cancer, an overdose of medication, or a condition that resulted in the development of drug dependency or drug abuse

### Study designs

Only four studies were randomised controlled trials (RCTs) [31, 35, 36, 43]. Most of the included studies were observational (11 studies), three of these prospective [32, 38, 42], seven retrospective [33, 34, 37, 39, 41, 44, 45], and one cross-sectional [40]. Data on outcomes was extracted for the end of the follow-up period of each included study.

### Participants

In ten studies at least 80% of participants were aged 65 years or older [32, 34–40, 43, 44]. In these studies, the lowest mean age was 69.3 years and the highest 80.5 years. The remaining five studies provided subgroup analyses of older people (≥65 or ≥71 or >80 years) [31, 33, 41, 42, 45]. Length of follow-up varied from none (cross-sectional study) [40] to 11 years [38].

Representation of males ranged from 24.1% [44] to 97% [41]. Eight studies reported ethnicity with most participants being white/Caucasian (up to 81%) [37]. Participants from four different continents were involved in the studies including America (*n* = 8), Europe (*n* = 7), Asia (*n* = 3) and Australia (*n* = 2). Comorbidities were reported in 11 studies, and congestive heart failure, myocardial infarction, hypertension, chronic kidney disease and hypothyroidism were the most commonly reported. Functional status was reported in one study [37]. No studies reported on the frailty level of the participants. Cognitive status was reported in four studies [37–40]. The percentage of participants with dementia was low in most studies ranging from 2.8% [38] to 67% [37]. Participant characteristics are summarised in Additional file 3: Table S1.

### Covariates in models

Adjustment for relevant covariates is important in observational studies to reduce confounder bias. The use of covariates indicates that a study’s authors have considered this issue, although by no means implies that bias has been eliminated. Many of the studies used long lists of covariates, therefore Additional file 3: Table S2 is given for online publication.

### Interventions and outcomes

Most of the included studies investigated the effect of metformin as monotherapy [33, 37, 39–42, 44, 45]. We classified comparison treatments as either “non-specific active treatments” (the comparator was not a single specific drug or treatment e.g. usual care, no insulin sensitizer, not on metformin), or as “specific active treatments” (the comparator was a specified treatment such as insulin, sulfonylureas, dipeptidyl peptidase-4 inhibitors, thiazolidinediones, and other specific drugs and combinations).

One RCT addressed both tier 1 and tier 2 outcomes [31]; the remaining RCTs investigated tier 1 outcomes only [35, 36, 43]. All the included observational studies addressed tier 2 outcomes only [32–34, 37–42, 44, 45]. We did not find any studies reporting on the following relevant endpoints: quality of life, life expectancy, functional impairment or status, and renal failure.

### Main findings

Table 2 summarises the results for each study for both metformin and comparison groups, with estimated risk ratios with 95% confidence intervals, together with statistical comparisons from the study. The results are organised by type of outcome (safety or efficacy), and then by type of comparator within each type of outcome (metformin against non-specific active treatments; metformin against other specific active treatments). A Additional file 3: Table S2 is available with the covariates that were taken into account in the statistical models of the included studies.

**Table 2:** Summary of study findings

Authors and publication year	Outcomes	Metformin cases/n^a^ (%)	Comparator cases/n^a^ (%)	Unadjusted Risk ratio^b^ (95% CI)	Reported Statistical comparison^c^	Result favours
Tier 1 outcomes (hypoglycaemia and adverse events): comparisons against other non-specific treatments						
Cryer 2005 [31]		Metformin	Usual care			
Subgrou*p* ≥ 65 years	Any SAE	371/2515 (14.8)	93/569 (16.3)	0.90 (0.73, 1.11)	NR	M
Tier 1 outcomes (hypoglycaemia and adverse events): comparisons against other specific treatments						
Janka 2007 [35]		Insulin glargine + OAD (glimepiride and metformin)	Premixed insulin			
			(*n* = 63 patients)			
	Confirmed + unconfirmed hypoglycaemia	(*n* = 67 patients)				
		5.6 events per p-yr.	11.4 events per p-yr	0.49 (0.41, 0.59)	*p* = 0.01	M
	Confirmed hypoglycaemia	3.7 events per p-yr	9.1 events per p-yr		*p* = 0.008	M
	Confirmed symptomatic hypoglycaemia	2.2 events per p-yr	5.0 events per p-yr	0.40 (0.33, 0.50)	*p* = 0.06	M
	Confirmed nocturnal hypoglycaemia	0.4 events per p-yr	0.7 events per p-yr	0.44 (0.33, 0.59)	*p* = 0.26	M
	Severe hypoglycaemia	0.0 events per p-yr	0.1 events per p-yr		*p* = 0.21	M
	One or more treatment-emergent AEs	32/67 (47.8)	27/63 (42.9)	0.55 (0.27, 1.12)		
				0.14 (0.01, 2.61)	NR	C
				1.11 (0.76, 1.63)		
Josephkutty et al. 1990 [36]		Metformin	Tolbutamide		NR	C
	Side effects	32 side effects reported by 21 patients	15 side effects reported by 20 patients			
Schweizer et al. 2009 [43]		Metformin	Vildagliptin			
	AEs	83/165 (50.3)	74/167 (44.3)	1.14 (0.90, 1.43)	NR	C
	SAEs	6/165 (3.6)	5/167 (3.0)	1.21 (0.38, 3.90)	NR	C
	Gastrointestinal AEs	41/165 (24.8)	25/167 (15.0)	1.66 (1.06, 2.60)	NR	C
	Hypoglycaemia	2/165 (1.2)	0/167 (0.0)	5.06 (0.24, 104.61)	NR	C
Tier 2 outcomes: comparisons against other non-specific treatments						
Cryer 2005 [31]		Metformin	Usual care			
	All-cause mortality	60/2515 (2.4)	12/569 (2.1)	1.13 (0.61, 2.09)	*p* = 0.878	C
Subgrou*p* ≥ 65 years	All-cause hospitalisations	334/2515 (13.3)	88/569 (15.5)	0.86 (0.69, 1.07)	*p* = 0.178	M
Inzucchi 2005 [34]		Metformin	No insulin sensitizer			
	1-year mortality	246/1273 (19.3)	2014/6641 (30.3)	0.64 (0.57, 0.72)	HR = 0.92 (0.81, 1.06)	M
	1-year MI readmission	210/1273 (16.5)	1247/6641 (18.8)	0.88 (0.77, 1.00)	HR = 1.02 (0.86, 1.20)	C
	1-year HF readmission	435/1273 (34.2)	2859/6641 (43.1)	0.79 (0.73, 0.86)	HR = 1.06 (0.95, 1.18)	C
	1-year all-cause readmission	759/1273 (59.6)	4268/6641 (64.3)	0.93 (0.88, 0.97)	HR = 1.04 (0.96, 1.13)	C
Masoudi 2005 [39]		Metformin	No insulin sensitizer			
	Mortality	460/1861 (24.7)	4345/12069 (36.0)	0.69 (0.63, 0.75)	HR = 0.87 (0.78, 0.97)	M
	All-cause readmission	1265/1861 (68.0)	8702/12069 (72.1)	0.94 (0.91, 0.97)	HR = 0.94 (0.89, 1.01)	M
	HF readmission	1091/1861 (58.6)	7821/12069 (64.8)	0.90 (0.87, 0.94)	HR = 0.92 (0.86, 0.99)	M
	Readmission for metabolic acidosis	2.3%	2.6%	*P* = 0.40		
MacDonald 2010 [38]		Metformin	No antidiabetic drugs			
	Mortality	155/376 (41)	733/1306 (56)	0.73 (0.65, 0.84)	OR = 0.65 (0.48, 0.87)	M
Moore et al. 2013 [40]		Metformin	Not on metformin			
		(*n* = 35 patients)	(*n* = 91 patients)			
	Cognitive performance	NR	NR		OR = 1.75 (0.81, 3.78)	C
					*p* = 0.158	
Roussel et al. 2010 [42]	Mortality:	Metformin	No metformin			
	Patients 65–80 years	191/3791 (5.0)	532/6768 (7.9)	0.64 (0.55, 0.75)	HR = 0.77 (0.62, 0.95), *p* = 0.02	M
	Patients >80 years	71/598 (11.9)	220/1492 (14.7)	0.81 (0.63, 1.03)	HR = 0.92 (0.66, 1.28), *p* = 0.61	M
Tier 2 outcomes: comparisons against other specific treatments						
Evans 2010 [32]		Metformin monotherapy + combination (*n* = 205)	SU monotherapy			
			(*n* = 217)			
	1-year mortality	NR	NR		OR = 0.60 (0.37, 0.97)	M
	Long-term mortality	NR	NR		OR = 0.67 (0.51, 0.88)	M
Roumie 2012 [41]		Metformin	SU			
		(*n* = 64,009 patients)	(*n* = 54,005 patients)			
Subgrou*p* ≥ 65 years	Hospitalization for acute MI, stroke or death	15.9 per 1,000p–yrs	24.6 per 1,000p–yrs		HR = 0.85 (0.78, 0.92)	M
	Hospitalization for acute MI or stroke	12.9 per 1,000p–yrs	18.5 per 1,000p–yrs		HR = 0.88 (0.81, 0.97)	M
Lapane 2015 [37]	Hospitalisation for hypoglycaemia	Metformin monotherapy	SU monotherapy			
		(*n* = 6151)	(*n* = 5807)			
	All ages	132 in 6518 p-yrs	289 in 6307 p-yrs	0.43 (0.35, 0.53)	HR = 0.42 (0.33, 0.53)	M
	Age 75–84	55 in 2524 p-yrs	104 in 2455 p-yrs	0.51 (0.37, 0.71)	HR = 0.50 (0.34, 0.73)	M
	Age 85+	39 in 2248 p-yrs	100 in 2167 p-yrs	0.38 (0.26, 0.54)	HR = 0.38 (0.25, 0.58)	M
	Hospitalisation for fractures related to falls					
	All ages	180 in 6305 p-yrs	194 in 6174 p-yrs	0.94 (0.76, 1.15)	HR = 0.88 (0.69, 1.12)	M
	Age 75–84	70 in 2478 p-yrs	86 in 2375 p-yrs	0.78 (0.57, 1.07)	HR = 0.73 (0.50, 1.05)	M
	Age 85+	74 in 2142 p-yrs	65 in 2114 p-yrs	1.12 (0.81, 1.57)	HR = 1.05 (0.68, 1.59)	C
	Falls					
	All ages	1844 in 4546 p-yrs	1864 in 4560 p-yrs	0.99 (0.93, 1.06)	HR = 1.02 (0.94, 1.11)	C
	Age 75–84	703 in 1785 p-yrs	756 in 1693 p-yrs	0.88 (0.80, 0.98)	HR = 0.90 (0.79, 1.02)	M
	Age 85+	697 in 1519 p-yrs	691 in 1547 p-yrs	1.03 (0.92, 1.14)	HR = 0.98 (0.86, 1.12)	M
Hung 2013 [33]		Metformin (*n* = 109)	Glyburide (*n* = 72)			
			47 in 181 p-yrs			
Subgrou*p* ≥ 71 years	Non-fatal CVD	30 in 414 p-yrs	Glimepiride (*n* = 50)	0.28 (0.18,0.44)	HR = 0.30 (0.18, 0.48)	M
	Non-fatal CVD	30 in 414 p-yrs	18 in 167 p-yrs	0.67 (0.38, 1.21)	NR	M
Inzucchi 2005 [34]		Metformin	Thiazolidinedione			
	Mortality	246/1273 (19.3)	237/819 (28.9)	0.67 (0.57, 0.78)	NR	M
	MI readmission	210/1273 (16.5)	154/819 (18.8)	0.88 (0.73, 1.06)	NR	M
	HF readmission	435/1273 (34.2)	402/819 (49.1)	0.70 (0.63, 0.77)	NR	M
	All-cause readmission	759/1273 (59.6)	555/819 (67.8)	0.88 (0.82, 0.94)	NR	M
Solomon 2009 [44]		Metformin	Thiazolidinediones			
	Fractures	110/4235 (2.6)	74/2347 (3.2) SU	0.82 (0.62,1.10)	RR = 0.76 (0.56, 1.02)	M
	Fractures	110/4235 (2.6)	480/13709 (3.5)	0.74 (0.60, 0.91)	NR	M
Tzoulaki 2009 [45]		Metformin monotherapy	1st generation SU monotherapy			
		>1.6mil intervals ^d^				
Subgrou*p* ≥ 65 years	MI	NR	NR		HR = 0.79 (0.65, 0.96)	M
	CHF	NR	NR		HR = 0.76 (0.68, 0.85)	M
	All-cause mortality	NR	NR		HR = 0.72 (0.67, 0.79)	M
			2nd generation SU monotherapy			
	MI	NR	NR		HR = 0.82 (0.74, 0.91)	M
	CHF	NR	NR		HR = 0.85 (0.79, 0.91)	M
	All-cause mortality	NR	NR		HR = 0.74 (0.70, 0.78)	M
			Rosiglitazone monotherapy			
	MI	NR	NR		HR = 0.85 (0.54, 1.33)	M
	CHF	NR	NR		HR = 0.93 (0.65, 1.33)	M
	All-cause mortality	NR	NR		HR = 0.98 (0.76, 1.27)	M
			Rosiglitazone combination			
	MI	NR	NR		HR = 0.81 (0.63, 1.06)	M
	CHF	NR	NR		HR = 0.76 (0.61, 0.93)	M
	All-cause mortality	NR	NR		HR = 1.10 (0.94, 1.28)	C
			Pioglitazone alone and combined			
	MI	NR	NR		HR = 1.23 (0.74, 2.08)	C
	CHF	NR	NR		HR = 0.90 (0.64, 1.26)	M
	All-cause mortality	NR	NR		HR = 1.54 (1.15, 2.04)	C
			Other drugs and combinations			
	MI	NR	NR		HR = 0.87 (0.77, 0.98)	M
	CHF	NR	NR		HR = 0.93 (0.85, 1.01)	M
	All-cause mortality	NR	NR		HR = 0.74 (0.70, 0.78)	M

*AEs* adverse events; *SAEs* serious adverse events; *MI* Myocardial Infarction; *CVD* cardiovascular disease; *CHF* congestive heart failure; *P*-*YRs* patient-years; *M* Metformin; *SU* Sulfonylureas; *C* Comparator; *CI* confidence interval; *HR* hazard ratio; *OR* Odds ratio; *RR* risk ratio; *NR* Not Reported

^a^number of patients with the outcome/total patients unless stated otherwise, unreported counts/rates were derived from available data where possible

^b^Calculated risk ratio unadjusted for covariates, zero cell adjustment applied where relevant

^c^Based on reported comparison adjusted for covariates, or if not reported the unadjusted risk ratio

^d^total treatment intervals across all treatments (metformin plus comparators) was >1.6 million, patients could have multiple intervals on different treatments

#### Tier 1 outcomes (hypoglycaemia and adverse events)

Tier 1 outcomes were investigated by all the included trials but none of the observational studies. In one large trial metformin was not significant different than usual care (non-specific active treatment) for serious adverse events [31]. Compared against other specific active treatments, a combination of insulin glargine plus glimepiride and metformin demonstrated significantly fewer hypoglycaemic events (both confirmed and unconfirmed hypoglycaemia) compared to premixed insulin [35]. However, in other trials, participants taking tolbutamide reported fewer side effects than participants on metformin [36] and vildagliptin outperformed metformin on all safety outcomes reported [43], but in neither case was any formal statistical comparison reported.

#### Tier 2 outcomes: Metformin compared to other non-specific active treatments

One trial and five observational studies compared metformin as monotherapy with non-specific active treatments for efficacy-related outcomes [31, 34, 38–40, 42]. Three large observational studies reported significantly fewer deaths in participants taking metformin compared to participants taking either no insulin sensitizer [39], no antidiabetic drugs [38], or no metformin [42]. However, there was no significant difference in mortality for patients aged over 80 years, and so was it for those patients with GFR ≤60 [42]. Inzucchi et al. (2005) [34] in another large study also reported that there was no significant difference in mortality in the metformin group. Admissions for various types of causes were evaluated by three studies [31, 34, 39] without significant differences between metformin and other active treatments, except for re-admission for heart failure which was significantly different favouring metformin [39].

#### Tier 2 outcomes: Metformin compared to other specific active treatments

Six observational studies compared metformin with specific active treatments on their effect on efficacy outcomes [32, 33, 37, 41, 44, 45]. One of these studies [32] reported substantially reduced mortality in the metformin monotherapy group (16% of the 422 participants) compared to groups taking metformin and sulfonylureas in combination (32%) and sulfonylurea monotherapy (51%). The remaining studies compared monotherapy with metformin against a range of other mostly monotherapy drug treatments. Metformin outperformed sulfonylureas with significantly fewer hospital admissions for acute myocardial infarction, stroke or death [41]; fewer hospitalisations for hypoglycaemia [37]; fewer events of non-fatal cardiovascular disease [33]; fewer fractures [44] and fewer events of myocardial infarction, congestive heart failure and all-cause mortality [45]. Other study results favoured metformin with significant differences in comparisons against thiazolidinediones [34] (mortality and all-cause and HF readmissions). There were no significant differences when metformin was compared to rosiglitazone and pioglitazone [45] (myocardial infarction, congestive heart failure and all-cause mortality).

### Excluded studies

Additional file 1 has the full list of reasons for exclusion of studies after full text analysis.

### Quality appraisal of included studies

#### Randomised trials

Four randomised trials were included and assessed for risk of bias (Table 3). Two of these trials did not provide enough information to assess the risk of bias [36, 43]. Another trial was judged to be of high risk of selection, performance and other bias [31]. One trial was judged to be of high risk of performance and detection bias [35].

**Table 3:** Quality appraisal for intervention studies

Source	Type of study	Selection bias		Performance bias	Detection bias	Attrition bias	Reporting bias	
		1. Random sequence generation	2. Allocation concealment	3. Blinding of participants and personnel	4. Blinding of outcome assessment	5. Incomplete outcome data	6. Selective reporting	7. Other bias
Cryer 2005 [31]	Randomised, open label, parallel-group, multicentre, clinical trial	UR	HR	HR	UR	LR	UR	HR
Janka 2007 [35]	Parallel-group, open-label, randomized, multinational clinical trial	LR	LR	HR	HR	LR	LR	LR
Josephkutty 1990 [36]	Randomized double-blind cross-over study	UR	UR	UR	UR	UR	UR	UR
Schweizer 2009 [43]	Randomized, active-controlled, parallel-group study	UR	UR	UR	UR	LR	UR	HR

*LR* low risk of bias; *HR* high risk of bias; *UR* unclear risk of bias

#### Observational studies

Quality appraisal was assessed with the CASP tool for the included 11 observational studies (Table 4). Most of the included studies reported sufficient detail to assess their quality. All studies addressed a clearly focused issue and all but one [33] used an appropriate method to answer their research question. Selection bias was not a problem in 8 of the 11 included studies as the recruitment method was adequate for the design (either cohort or case-control study) [32–34, 37–39, 44, 45]. Potential confounding factors were taken into account in the design or analysis in 10 of the 11 included studies [33, 34, 37–42, 44, 45]. It was clear that the results of all studies (apart from the cross-sectional study) could be applied to our population of interest (older people). Nearly half the studies did not report accurately how the exposure or outcome were measured, which could lead to high risk of measurement or classification bias [34, 40–42, 45]. For example, the comparison group was not clearly defined in one study [34]. It was unclear whether the follow-up was sufficiently long in 5 studies [32, 33, 39, 41, 42] and it was considered that the follow-up insufficient in 3 studies [34, 37, 44].

**Table 4:** Quality appraisal for observational studies according to the Critical Appraisal Skills Programme (CASP)

Source	Type of study	1. Focused issue	2. Appropriate method	3. Recruitment	4. Selection of controls	5. Exposure measured	6. Outcome measured	7. Confounding factors identified	8. Confounding design/analysis	9. Follow up complete	10. Follow up long	11. Results of this study	12. How precise results/risk estimate	13. Believe results	14. Results be applied	15. Results fit evidence
Evans 2010 [32]	Population-based cohort study	Y	Y	Y	NA	Y	Y	N	N	Y	U			Y	Y	U
Hung 2013 [33]	population-based retrospective cohort study	Y	U	Y	NA	Y	N	N	Y	U	U			Y	Y	Y
Inzucchi 2005 [34]	Retrospective cohort study	Y	Y	Y	NA	N	Y	Y	Y	Y	N			U	Y	U
Lapane 2015 [37]	Retrospective cohort study	Y	Y	Y	NA	Y	N	Y	Y	U	N			Y	Y	Y
MacDonald 2010 [38]	Case-control study	Y	Y	Y	Y	Y	Y	Y	Y	Y	Y			Y	Y	Y
Masoudi 2005 [39]	Retrospective cohort	Y	Y	Y	NA	Y	N	Y	Y	U	U			U	Y	U
Moore 2013 [40]	Cross sectional study	Y	Y	N	N	N	Y	U	Y	NA	NA			U	U	N
Roumie 2012 [41]	Retrospective cohort study	Y	Y	U	NA	U	U	Y	Y	U	U			U	Y	U
Roussel 2010 [42]	Prospective, observational registry	Y	Y	N	NA	U	Y	U	Y	Y	U			Y	Y	Y
Solomon 2009 [44]	Retrospective cohort	Y	Y	Y	NA	Y	Y	Y	Y	Y	N			U	Y	Y
Tzoulaki 2009 [45]	Retrospective cohort study	Y	Y	Y	NA	U	Y	Y	Y	Y	Y			U	Y	Y

*Y* yes; *N* no; *U* unclear; *NA* not applicable. Items 11 and 12 are part of the findings in Table 2

### Additional references of interest for the development of recommendations

One further reference was incorporated as additional reference of interest for the development of the recommendations [46]. A clinical guideline from the American Geriatrics Society which included recommendations about the management of DM2 in older people with renal insufficiency [46] was included as an additional reference of interest for the development of recommendations.

## Recommendations

Four recommendations about stopping the use of metformin in older people with DM2 (Table 5) were developed related to halting In order to discuss and agree on these recommendations three meetings took place between YVM (researcher) and ARG (researcher and clinician). IK (senior clinician and researcher) and AS (senior clinician and researcher) participated in one of these meetings. The whole body of evidence identified in the SR was taken into consideration for the development of the recommendations. However, each recommendation was specially supported by the following specific studies included in the SR or considered as additional references of interest: a clinical guideline, an observational study without high quality [42] and two randomised trials with insufficient information to assess their risk of bias [36, 43]. All recommendations were considered to be weak and based on evidence of low quality, and the reasons for this are reported in Table 5. The recommendations were included in the Comprehensive Medication Review (CMR) tool developed as part of the PRIMA-eDS project, and they were formulated according to their strength and the quality of their evidence [19].

**Table 5:** Recommendations to stop the use of metformin in older people with type 2 Diabetes mellitus

Recommendations	Strength of the recommendation	Quality of the evidence	Type of evidence
It is suggested to discontinue metformin for the management of type 2 diabetes mellitus in patients with 2 or more of the following risk factors: age > 80; gastrointestinal complaints during the last year; GFR ≤60 ml/min. The benefit of metformin in this patient is uncertain and it is possibly outweighed by the risk of adverse drug reactions, depending on their severity.	Weak	Low	Observational study [42]; RCTs [36, 43]; clinical guideline [46]
	Reason: uncertainty about the magnitude of the benefits and harms.	It was considered to downgrade the quality of the evidence to low quality because there were study limitations (1 observational study with limitations and 2 RCTs with unclear risk of bias), indirectness (observational study with subgroup analysis), inconsistency (different types of comparisons evaluated).	
It is suggested to discontinue metformin for the management of type 2 diabetes mellitus in patients 80 years and older taking the life expectancy, physical and functional status of the patient into account. Patients who are concerned about adverse events or appear to experience AE may reasonably choose not to take metformin.	Weak	Low	Observational study [42]
	Reason: uncertainty about the magnitude of the benefits and harms.	It was considered to keep the quality of the evidence as low quality because this observational study had limitations: data in older people was from subgroup analysis, lack of reporting on recruitment and confounding factors.	
It is suggested to discontinue metformin for the management of type 2 diabetes mellitus in patients with gastrointestinal complaints taking the possible benefit and the severity of the patient complaints as possible dverse drug reactions into account.	Weak	Low	RCTs [36, 43]
	Reason: small RCTs with low quality and no significant benefits with metformin; uncertainty about the magnitude of the benefits and harms.	It was considered to downgrade the quality of the evidence to low quality because there were study limitations (2 RCTs with unclear risk of bias) and inconsistency (different types of comparisons evaluated).	
It is suggested to discontinue metformin for the management of type 2 diabetes mellitus in patients with renal insufficiency because metformin may increase the risk of lactic acidosis.	Weak	Low	Clinical guideline [46]
	Reason: evidence from a clinical guideline; uncertainty about the magnitude of the benefits and harms.	It was considered to keep the quality of the evidence as low quality because it was from a clinical guideline.	

## Discussion

Our aim was to systematically review the existing evidence on the risks and benefits of the use of metformin for the management of DM2 in older people. We therefore included only those studies where a high proportion of participants were aged 65 years or older, as specified in our inclusion criteria. No systematic review or meta-analysis fulfilled our inclusion criteria, and we finally included 4 RCTs and 11 observational studies, with most observational studies being retrospective.

When comparing metformin with sulfonylureas, results suggest that metformin may be better than sulfonylureas in reducing several outcomes such as cardiovascular outcomes, mortality, hospitalisation for hypoglycaemia, or risk of falls in people aged 65 and older with DM2 [32, 33, 37, 41, 45]. When comparing metformin with no insulin sensitizer antidiabetic drugs, divergent results were found depending on the study population [34, 39] Generally, these results are in line with clinical guidelines recommending metformin as the first-line drug treatment for adults with DM2 [10]. Guidelines also suggest that if initial drug treatment with metformin fails to control levels of glycated haemoglobin, dual therapy should be considered [10]*.* Only one study was identified which specifically analysed risks and benefits of combining metformin with other antidiabetic drugs, where risk of hypoglycaemia with the combination of metformin, sulfonylurea and insulin glargine compared to premixed insulin was decreased [35]. Thus, there seems to be a lack of evidence analysing the benefits and risks of combined therapy including metformin in older people with DM2.

Few studies provided data on adverse events other than hypoglycaemia and falls with the use of metformin in this population such as bloatedness, nausea, and diarrhoea [31, 36, 39, 40, 43]. Furthermore, only two studies analysed the adverse event of lactic acidosis, and no increased risk was found for metformin [31, 39]. All these studies had considerable methodological limitations. We did not identify any study reporting on renal failure as adverse event of metformin. Thus, further prospective studies should evaluate the adverse events of the use of metformin in older people.

The benefits of metformin on the mortality of very old people (aged 80 years over) were investigated by only one included study [42]. Here, mortality was significantly decreased with the use of metformin in people aged 65–80 but the effect was not significant for the population aged 80 and older. The study could been underpowered for this subgroup analysis, but evidence this may also suggest that the benefits of metformin on mortality may be non-existent for very old people, especially those with limited life expectancy, as suggested by other authors [47].

The included studies rarely reported on the functional level and cognitive status of the participants; the use of concomitant drugs and the presence of other diseases were more frequently reported but focused mostly on cardiovascular drugs and diseases. Thus, the present systematic review demonstrates that not only very old people, but also cognitively and functionally impaired people and old people with multimorbidity are underrepresented or at least underreported in existing studies, which limits the generalisability of already scarce evidence for this heterogeneous group of older people. A growing body of literature presents functional and cognitive status as well as multimorbidity as predictors of mortality among older people independently from their chronological age [48–50], which supports the idea that further studies analysing these aspects are necessary.

Although the lack of evidence has been previously commented on by several authors [1, 47, 51], to the best of our knowledge, ours is the first study to systematically review all available evidence on the risks and benefits of metformin for the management of DM2 in older people.

Current guidelines suggest that for older people with limited life expectancy or functional limitation, intensive glycaemic control is not recommended [1, 47, 52]*.* However, older people who are functionally and cognitively intact and have significant life expectancy should be treated with goals similar to those developed for younger people [1]. In the present systematic review, six studies [32, 35, 36, 41, 43, 45] reported on mean baseline levels of glycated haemoglobin which ranged between 7.0% [41] and 10.2% [36]. One of these studies considered a glycated haemoglobin level of 7% or less without experiencing nocturnal hypoglycaemia to represent successful therapy [35].

None of the other studies reported their target glycaemic control level clearly. It would be useful for future studies on the management of DM2 to report the target glycaemic control level, especially for frail older people.

Our research team developed four recommendations using both the results of the systematic review and the additional references identified [36, 42, 43, 46]. The recommendations advise clinicians to consider discontinuing metformin in people aged 80 and older, those with gastrointestinal complaints during the last year, and/or those with Glomerular Filtration Rate (GFR) ≤60 ml/min. These recommendations have been incorporated in the trial version of an electronic decision support tool that aims to help general practitioners to reduce inappropriate prescriptions for older people with multimorbidity. Decisions on prescription or de-prescription of metformin should be made taking the symptoms and individual characteristics of each patient into account and clinicians receive instructions on that. Currently, the tool is being tested in a cluster randomised controlled trial in four European countries [53].

This systematic review has limitations. The search strategy and inclusion criteria were designed to identify studies focusing on older people; studies on the general population that may have contained relevant information for the older population might have been overlooked. However, using independent reviewers for study selection and our peer reviewed process of development of recommendations should have minimised this problem. Our recommendations focus only on the discontinuation of metformin, as it was not the aim of the PRIMA-eDS project to develop recommendations when to use metformin. Nevertheless, this systematic review aims at providing an overview of the existing evidence on both the benefits and risks of the use of metformin in older people.

## Conclusions

This study highlights the lack of good quality evidence on the risks and benefits of metformin for the management of DM2 in older people. The use of metformin seems associated with benefits to lower mortality risk in older people, and may also be associated with a reduced risk of adverse events such as hypoglycaemia and non-fatal cardiovascular events, than other antidiabetic drugs, especially sulfonylureas. However, no prospective studies focussing on very old (80 and older) and functionally and cognitively impaired older people are available. In very old people, those with renal insufficiency (GFR ≤60 ml/min) and those with gastrointestinal complaints during the last year, the discontinuation of metformin should be considered, especially for those with limited life expectancy or functional impairment. There is an urgent need for studies on the risks and benefits of metformin for the management of DM2 in these populations in order to guide clinicians in planning of individualised patient care.

## Additional files

Additional file 1: Metformin diabetes older adults SR. (XLSX 298 kb)

Additional file 2: Metformin diabetes older adults SR. (DOCX 185 kb)

Additional file 3: Supplementary tables Metformin diabetes older adults SR. (DOCX 83 kb)

## Abbreviations

AMSTAR: A measurement tool to assess systematic reviews
CASP: Critical Appraisal Skills Programme
CMR: Comprehensive Medication Review
DARE: Database of Abstracts or Reviews of Effects
DM2: Type 2 diabetes mellitus
GFR: Glomerular filtration rate
GRADE: Grading of Recommendations Assessment, Development and Evaluation
HTA: Health Technology Assessment
IPA: International Pharmaceutical Abstracts
PICOS: Population, intervention, comparison, outcomes and study design
PRIMA-eDS: Polypharmacy in chronic diseases: Reduction of Inappropriate Medication and Adverse drug events in elderly populations by electronic Decision Support
PRISMA: Preferred Reporting Items for Systematic Reviews and Meta-Analyses
RCTs: Randomised controlled trials
SR: Systematic review
