# Supplementary material for: Efficacy and safety of metformin in the management of type 2 diabetes mellitus in older adults: a systematic review for the development of recommendations to reduce potentially inappropriate prescribing
Source: BMC Geriatr. 2017 Oct 16;17(Suppl 1):227. doi: 10.1186/s12877-017-0574-5 (PMC5647555; doi:10.1186/s12877-017-0574-5)
Supplement: Supplementary file 3 — Supplementary tables Metformin diabetes older adults SR. (DOCX 83 kb) [file 12877_2017_574_MOESM3_ESM.docx]

**Supplementary tables**

**Table S1: Characteristics of participants in included studies**

ACE-I: angiotensin-converting-enzyme inhibitor, ARBs: Angiotensin II Receptor Blockers, CCB: Calcium channel blocker, CVD: cardiovascular disease, DM2: type 2 diabetes mellitus, P: participants, SD: standard deviation, SU: sulfonylurea, TZD: thiazolidinedione, UK: United Kingdom, * Inzucchi (2005) [[34](#_ENREF_34)] provided a long list of comorbidities and concomitant medications but we are only reporting the most frequent comorbidities and concomitant medications.

^a^ mean age (standard deviation), ^b^ median age (range), ^c^ median age (interquartile range)

| **Authors and publication year** | **Setting / country / ethnicity** | **Male sex** | **Age** | **Reported comorbidities** | **Reported concomitant medications** | **Functional status / Frailty level** | **Cognitive status** |
| --- | --- | --- | --- | --- | --- | --- | --- |
| Cryer 2005 [[31](#_ENREF_31)] | P using metformin:  White / Caucasian: 78%  African American: 16.2%  Asian: 2.9%  Other: 2.8%  P under usual care:  White / Caucasian: 76.2%  African American: 17.5%  Asian: 2.7%  Other: 3.6% | P using metformin: 49.3%  P under usual care: 49.5% | P using metformin: 58.3 (12.9)^a^  P under usual care: 58.8 (13.1)^a^ | Not reported | Not reported | Not reported | Not reported |
| Evans 2010 [[32](#_ENREF_32)] | Scotland | P using SU: 54.8%  P using any metformin (alone/combination): 52.7% | P using SU: 76.7 (9.8)^a^  P using any metformin (alone/combination): 74.1 (8.8)^a^ | Not reported | P using SU:  ACE-I: 32.6%  Aspirin: 51.6%  Diuretic: 21.9%  β-blocker: 11.6%  P using any metformin:  ACE-I: 42.9%  Aspirin: 68.0%  Diuretic: 16.7%  β-blocker: 16.7% | Not reported | Not reported |
| Hung 2013 [[33](#_ENREF_33)] | Taiwan. | P using glyburide: 56.4%  P using glimepiride: 55.6%  P using metformin: 46.2% | P using glyburide: 60.5 (11.4)^a^  P using glimepiride: 59.2 (11.7)^a^  P using metformin: 58.7 (11.9)^a^ | Hypertension:  P using glyburide: 64.5 %  P using glimepiride: 64.1%  P using metformin: 66.4%  Dyslipidaemia:  P using glyburide: 29.4%  P using glimepiride: 38.9%  P using metformin: 43.5% | P using glyburide: CCB: 40.0%  Statin: 12.1%  Anti-platelet medicines: 13.9%  Anti-coagulant medicines: 0.3%  P using glimepiride:  CCB: 44.9%  Statin: 17.9%  Anti-platelet medicines: 20.5%  P using metformin:  CCB: 38.7%  Statin: 30.1%  Anti-platelet medicines: 20.8% | Not reported | Not reported |
| Inzucchi 2005 [[34](#_ENREF_34)]* | USA | P using no insulin sensitizer: 46.8%  P using metformin: 51.8%  P using TZD: 47.0%  P using metformin + TZD: 53.2% | P using no insulin sensitizer: 76.8 (7.1)^a^  P using metformin: 75.2 (7.0)^a^  P using TZD: 75.8 (6.9)^a^  P using metformin + TZD: 73.6 (6.8)^a^ | P using no insulin sensitizer:  Congestive heart failure: 41.6%  MI: 44.5%  Hypertension: 80.3%  P using metformin:  Congestive heart failure: 28.1%  MI: 39.1%  Hypertension: 77.6%  P using TZD:  Congestive heart failure: 46.6%  MI: 52.0%  Hypertension: 80.7%  P using metformin + TZD:  Congestive heart failure: 30.9%  MI: 50.4%  Hypertension: 76.3% | P using no insulin sensitizer:  ACE-I: 54.8%  β-blocker: 63.2%  CCB: 25.8%  Aspirin: 76.0%  Statin: 32.1%  P using metformin:  ACE-I: 59.9%  β-blocker: 68.8%  CCB: 23.6%  Aspirin: 79.7%  Statin: 42.3%  P using TZD:  ACE-I: 54.5%  β-blocker: 66.4%  CCB: 27.6%  Aspirin: 75.7%  Statin: 48.0%  P using metformin + TZD:  ACE-I: 71.9%  β-blocker: 74.1%  CCB: 18.0%  Aspirin: 77.0%  Statin: 57.6% | Not reported | Not reported |
| Janka 2007 [[35](#_ENREF_35)] | 66 sites in 10 European countries: Austria, Finland, France, Germany, Italy, The Netherlands, Spain, Sweden, Switzerland, United Kingdom. | P using insulin glargine + glimepiride and metformin: 64%  P using premixed insulin: 48% | P using insulin glargine + glimepiride and metformin: 69.3 (2.8)^a^  P using premixed insulin: 69.6 (4.1)^a^ | Not reported | Not reported | Not reported | Not reported |
| Josephkutty 1990 [[36](#_ENREF_36)] | Diabetic clinic / UK | P using metformin / tolbutamide: 30%  P using tolbutamide / metformin: 30% | P using metformin / tolbutamide: 76.5 (67-94)^b^  P using tolbutamide / metformin: 80.5 (72-90)^b^ | Not reported | Not reported | Not reported | Not reported |
| Lapane 2015 [[37](#_ENREF_37)] | Nursing home residents  P using SU:  White 80.6%  Black 13.6%  Other 6.0%  P using biguanide:  White 81.0%  Black 13.0%  Other 6.3% | P using SU: 30.5%  P using biguanide: 30.5% | Overall: 91% ≥65 years  P using SU: 88% ≥65 years  P using biguanide: 94% ≥65 years | P using biguanide:  Hypertension: 79.4%  Congestive heart failure: 27.3%  Chronic kidney disease: 29.3%  Hypothyroidism: 21.4%  Atherosclerotic heart disease: 16.7%  Osteoporosis: 18.5%  Stroke or TIA: 20.0%  P using SU:  Hypertension: 79.3%  Congestive heart failure: 30.9%  Chronic kidney disease: 28.7%  Hypothyroidism: 21.8%  Atherosclerotic heart disease: 17.4%  Osteoporosis: 18.5%  Stroke or TIA: 19.9% | P using biguanide:  Oral glucocorticoid use: 20,9%  Biphosphonates: 7,7%  Parathyroid hormone: < 0,2%  Calcitonin: 2,6%  Estrogen: 1,8%  P using SU:  Oral glucocorticoid use: 19,3%  Biphosphonates: 9,4%  Parathyroid hormone: < 0,2%  Calcitonin: 1,9%  Estrogen: 2,8%  For the other concomitant drugs (benzodiazepins, antidepressants, antipsychotics) no more details are reported. | Dependent in activities of daily living :  P using SU: 41.6%  P using biguanide: 41.7% | Dementia, including Alzheimer´s disease:  P using SU: 65.2%  P using biguanide: 67.4%  Severe cognitive impairment:  P using SU: 9.5%  P using biguanide: 9.2% |
| MacDonald 2010 [[38](#_ENREF_38)] | General practice in the UK | Controls (P with DM2and heart failure alive): 53.2%  Cases (P with DM2 and heart failure who died): 53.2% | 78 (8)^a^  Controls:  77.8 (7.8)^a^, 95% ≥65 years  Cases:  78.2 (8.0)^a^, 95% ≥65 years | Controls:  Hypertension: 56.9%  Prior MI or coronary revascularisation: 30.4%  Angina: 41.6%  Atrial Fibrillation: 36.0%  Cerebrovascular disease: 23.3%  Cases:  Hypertension: 52.5%  Prior MI or coronary revascularisation: 38.3%  Angina: 41.2%  Atrial Fibrillation: 36.1%  Cerebrovascular disease: 29.9% | Controls:  ACE-I: 60.9%  ARBs: 13.1%  Aspirin: 53.8%  Digoxin: 33.0%  β-blocker: 27.1%  Statin: 46.5%  Spironolactone: 11.9%  Cases:  ACE-I: 47.6%  ARBs: 7.1%  Aspirin: 42.0%  Digoxin: 28.4%  β-blocker: 19.2%  Statin: 35.0%  Spironolactone: 16.1% | Not reported | Dementia:  Controls: 2.8%  Cases: 4.2% |
| Masoudi 2005 [[39](#_ENREF_39)] | The National Heart Care Project (Medicare & Medicaid Services initiative) database of patient/ USA | No insulin sensitizer: 42%  TZD: 39%  Metformin: 43% | 76.4 (7.1)^a^ | No insulin sensitizer:  Hypertension: 70%  Coronary artery disease 66%  MI: 34%  Cerebrovascular accident: 22%  Chronic lung disease: 34%  TZD:  Hypertension: 72%  Coronary artery disease 68%  MI: 32%  Cerebrovascular accident: 20%  Chronic lung disease: 34%  Metformin:  Hypertension: 71%  Coronary artery disease 61%  MI: 31%  Cerebrovascular accident: 18%  Chronic lung disease: 33% | Discharge medication:  No insulin sensitizer:  ACE-I: 57%  β-blocker: 32%  Aspirin: 44%  HMG-CoA reductase inhibitor: 21%  SU: 51%  Insulin: 55%  TZD:  ACE-I: 54%  β-blocker: 37%  Aspirin: 44%  HMG-CoA reductase inhibitor: 34%  SU: 39%  Insulin: 47%  Metformin:  ACE-I: 64%  β-blocker: 38%  Aspirin: 44%  HMG-CoA reductase inhibitor: 27%  SU: 56%  Insulin: 19% | Not reported | Dementia:  No insulin sensitizer: 8%  TZD: 6%  Metformin: 6% |
| Moore 2013 [[40](#_ENREF_40)] | Australia | Overall 40.5%  In DM2 subgroup: 46.8% | Overall 73.8 (8.3)^a^ | Depression: 31.7% | Calcium supplements: 35% in DM2 subgroup | Not reported | Overall:  Not impaired: 50.4%  Minimally impaired: 21.8%  Mildly impaired 17.7%  Most impaired 10.1% |
| Roumie 2012 [[41](#_ENREF_41)] | Primary care / Nashville, Tennesse | Overall 97%  Metformin group 95%  SU group 97% | Overall 64 (54-76) ^c^  metformin group: 62 (56-71) ^c^  SU group:67 (57-76) ^c^ | P using metformin:  MI/coronary disease: 20%  Stroke/TIA/carotid revascularization: 8%  Peripheral artery disease: 3%  COPD/emphysema: 9%  Atrial fibrillation/flutter: 3%  P using SU:  MI/coronary disease: 23%  Stroke/TIA/carotid revascularization: 9%  Peripheral artery disease: 3%  COPD/emphysema: 9%  Atrial fibrillation/flutter: 4% | P using metformin:  ACE-I or ARBs: 58%  β-blocker: 36%  Calcium-channel blockers: 23%  Other antihypertensive: 16%  Statins: 61%  Other lipid-lowering agents: 13%  Antiarrhythmics: 1%  Anticoagulants: 4%  Antipsychotics: 7%  Digoxin: 3%  Thiazides and other diuretics: 33%  Loop diuretics: 9%  Nitrates: 11%  Aspirin: 17%Platelet inhibitors: 6%  P using SU:  ACE-I or ARBs: 57%  β-blocker: 38%  CCB: 25%  Other antihypertensives: 18%  Statins: 55%  Other lipid-lowering agents: 11%  Antiarrhythmics: 1%  Anticoagulants: 6%  Antipsychotics: 7%  Digoxin: 6%  Thiazides and other diuretics: 30%  Loop diuretics: 14%  Nitrates: 14%  Aspirin: 17%  Platelet inhibitors: 8% | Not reported | Not reported |
| Roussel 2010 [[42](#_ENREF_42)] | Outpatients / 44 countries from North America, Latin America, Western Europe, Eastern Europe, Middle East, Asia, Australia, and Japan | P using metformin: 65.5%  P without metformin: 65.4% | P using metformin: 67.1 (9.3)^a^  P without metformin: 69.2 (9.5)^a^ | P using metformin: Hypertension: 87.7%  Prior congestive heart failure: 16.5%  P without metformin:  Hypertension: 86.6%  Prior congestive heart failure: 23.0% | P using metformin:  Acetylsalicylic acid: 73.3%  Statins: 75.2%  ARBs: 25.2%  ACE-I: 54.3%  β-blocker: 53.9%  CCB: 36.2%  Diuretics: 45.4%  Nitrates: 29.3%  SU: 49.6%  TZD: 17.1%  Insulin: 17.3%  P without metformin:  Acetylsalicylic acid: 68.7%  Statins: 74.0%  ARBs: 25.5%  ACE-I: 48.5%  β-blocker: 50.6%  CCB: 37.5%  Diuretics: 48.2%  Nitrates: 31.9%  SU: 39.0%  TZD: 13.4%  Insulin: 35.0% | Not reported | Not reported |
| Schweizer 2009 [[43](#_ENREF_43)] | 14 countries in Europe, the Americas and Asia | P using metformin: 53.0%  P using vildagliptin: 44.4% | P using metformin: 70.2 (5.1) ^a^  P using vildagliptin: 71.6 (5.2) ^a^ | Overall sample:  Hypertension: 70%  Dyslipidaemia: 40%  Mild renal insufficiency: 50%  Previous cardiac disorders: 33% | Overall sample:  ACE-I: 37%  β-blocker: 28%  CCB: 20%  ARBs: 19%  Lipid lowering agents: 39%  Platelet aggregation inhibitors: 33% | Not reported | Not reported |
| Solomon 2009 [[44](#_ENREF_44)] | USA | Overall: 24.1%  Metformin monotherapy: 21.0%  TZD monotherapy: 23.6%  SU monotherapy: 24.7% | Metformin monotherapy: 76 (7)^a^  TZD monotherapy: 77 (7)^a^  SU monotherapy: 78 (7)^a^ | Metformin monotherapy: Diabetes-related Renal disease: 1%  Retinopathy: 2.6%  Peripheral neuropathy: 4.7%  Osteoporosis: 8.7%  Hyperparathyroidism: 3.6%  Rheumatoid arthritis: 2.2%  Congestive heart failure: 3.5%  TZD monotherapy: Diabetes-related Renal disease: 2.47%  Retinopathy: 3.5%  Peripheral neuropathy: 6.6%  Osteoporosis: 7.3%  Hyperparathyroidism: 1%  Rheumatoid arthritis: 2.2%  Congestive heart failure: 6.2%  SU monotherapy: Diabetes-related Renal disease: 1.8%  Retinopathy: 3.1%  Peripheral neuropathy: 5.6%  Osteoporosis: 6.3%  Hyperparathyroidism: 1%  Rheumatoid arthritis: 2.5%  Congestive heart failure: 7.6% | Metformin monotherapy:  Oral glucocorticoid use: 6.8%  Osteoporosis medication: 13.3%  No. of different medications: 7 (SD 4)  TZD monotherapy:  Oral glucocorticoid use: 8.2%  Osteoporosis medication: 12.4%  No. of different medications: 8 (SD 4)  SU monotherapy:  Oral glucocorticoid use: 7.0%  Osteoporosis medication: 8.9%  No. of different medications: 8 (SD 4) | Not reported | Not reported |
| Tzoulaki 2009 [[45](#_ENREF_45)] | General practice research databases / UK | First generation SU: 48.6%  Second generation SU: 52.6%  Rosiglitazone: 50.5%  Rosiglitazone combination: 53.6%  Pioglitazone monotherapy or combination: 54.3%  Other drugs or combinations: 53.6%  Metformin: 50.6% | 65.0 (11.9)^a^ | First generation SU: CVD: 15.0%  Peripheral arterial disease: 3.5%  Second generation SU: CVD: 16.5%  Peripheral arterial disease: 2.8%  Rosiglitazone: CVD: 15.5%  Peripheral arterial disease: 3.0%  Rosiglitazone combination: CVD: 14.5%  Peripheral arterial disease: 2.9%  Pioglitazone monotherapy or combination: CVD: 15.4%  Peripheral arterial disease: 2.5%  Other drugs or combinations: CVD: 17.6%  Peripheral arterial disease: 3.4%  Metformin: CVD: 13.4%  Peripheral arterial disease: 2.1% | First generation SU: Aspirin: 16.6%  Statin or fibrate: 8.8%  ACE-I or ARBs: 18.4%  NSAIDS: 25.2%  Second generation SU: Aspirin: 17.7%  Statin or fibrate: 18.2%  ACE-I or ARBs: 24.5%  NSAIDS: 24.5%  Rosiglitazone: Aspirin: 14.5%  Statin or fibrate: 28.5%  ACE-I or ARBs: 26.4%  NSAIDS: 18.7%  Rosiglitazone combination: Aspirin: 29.8%  Statin or fibrate: 50.9%  ACE-I or ARBs: 45.0%  NSAIDS: 36.2%  Pioglitazone monotherapy or combination: Aspirin: 24.9%  Statin or fibrate: 40.8%  ACE-I or ARBs: 38.0%  NSAIDS: 30.7%  Other drugs or combinations: Aspirin: 23.8%  Statin or fibrate: 28.4%  ACE-I or ARBs: 33.6%  NSAIDS: 31.2%  Metformin: Aspirin 20.1%  Statin or fibrate 29.2%  ACE-I or ARBs: 30.3%  NSAIDS 26.6% | Not reported | Not reported |

**Table S2: Covariates in the included studies' models**

| **Authors and publication year** | **Type of reported adjusted outcome^[[1]](#endnote-1)^** | **Covariates in model** |
| --- | --- | --- |
| Cryer 2005 [[31](file:///C:\Users\lschlender\AppData\Local\Microsoft\Windows\Temporary%20Internet%20Files\Content.Outlook\4XGBQ7N7\Metformin%20diabetes%20older%20adults%20systematic%20review_clean_ARG.docx#_ENREF_31)] | No adjusted outcomes | - |
| Evans 2010 [[32](file:///C:\Users\lschlender\AppData\Local\Microsoft\Windows\Temporary%20Internet%20Files\Content.Outlook\4XGBQ7N7\Metformin%20diabetes%20older%20adults%20systematic%20review_clean_ARG.docx#_ENREF_32)] | Adjusted odds ratios | Sex, age, duration, previous hospital admission (yes vs no), creatinine (quartile 1-4), hemoglobin A1c, angiotensin-converting-enzyme inhibitor (yes vs no), aspirin (yes vs no), diuretic (yes vs no), ß-blocker (yes vs no) |
| Hung 2013 [[33](file:///C:\Users\lschlender\AppData\Local\Microsoft\Windows\Temporary%20Internet%20Files\Content.Outlook\4XGBQ7N7\Metformin%20diabetes%20older%20adults%20systematic%20review_clean_ARG.docx#_ENREF_33)] | Adjusted hazards ratios | Sex, age, angiotensin-converting-enzyme inhibitor, angiotensin receptor blocker, calcium channel blocker, β-blocker, statin, anti-platelet medicine, anticoagulant medicine |
| Inzucchi 2005 [[34](file:///C:\Users\lschlender\AppData\Local\Microsoft\Windows\Temporary%20Internet%20Files\Content.Outlook\4XGBQ7N7\Metformin%20diabetes%20older%20adults%20systematic%20review_clean_ARG.docx#_ENREF_34)] | Adjusted hazards ratios | Age, sex, and race, history of heart failure, myocardial infarction, hypertension, and revascularization, admission source, mobility, cerebral vascular accident, chronic pulmonary disease, urinary incontinence, and dementia, systolic blood pressure, respiratory rate, heart failure, sodium, glucose, blood urea nitrogen, creatinine, white blood cell count, and hematocrit, atrial fibrillation, heart failure/pulmonary edema on admission chest radiograph, cardiac catheterization, percutaneous coronary intervention, coronary artery bypass grafting, and diabetes complications, sulfonylureas and non-sulfonylurea insulin secretagogues, glucosidase inhibitors, and insulin, aspirin, angiotensin-converting-enzyme inhibitors, adrenergic receptor blockers, calcium channel blockers, statins, and fibrates, admission blood glucose level, prescription of other antihyperglycemic drugs at hospital discharge, the presence of the ICD-9 code for a diabetes complication, diabetic nephropathy, diabetic retinopathy, diabetic neuropathy, and peripheral vascular disease, attending physician (specialty and board certification), treating hospital (bed size, geographic location, ownership, level of cardiac care facilities, teaching status), the models were also adjusted for clustering of patients by hospitals |
| Janka 2007 [[35](file:///C:\Users\lschlender\AppData\Local\Microsoft\Windows\Temporary%20Internet%20Files\Content.Outlook\4XGBQ7N7\Metformin%20diabetes%20older%20adults%20systematic%20review_clean_ARG.docx#_ENREF_35)] | Adjusted mean differences | Hemoglobin A1c from baseline (before insulin initiation) |
| Josephkutty 1990 [[36](file:///C:\Users\lschlender\AppData\Local\Microsoft\Windows\Temporary%20Internet%20Files\Content.Outlook\4XGBQ7N7\Metformin%20diabetes%20older%20adults%20systematic%20review_clean_ARG.docx#_ENREF_36)] | No adjusted outcomes | - |
| Lapane 2015 [[37](file:///C:\Users\lschlender\AppData\Local\Microsoft\Windows\Temporary%20Internet%20Files\Content.Outlook\4XGBQ7N7\Metformin%20diabetes%20older%20adults%20systematic%20review_clean_ARG.docx#_ENREF_37)] | Adjusted hazards ratios | Age, race/ethnicity, sex, body mass index, concomitant medications (e.g., benzodiazepines, antidepressants, antipsychotics, bisphosphonates and other drugs that may strengthen or weaken bones), comorbid conditions (e.g., osteoporosis, dementia, stroke), sensory, cognitive, or physical functioning impairments, and diabetic retinopathy |
| MacDonald 2010 [[38](file:///C:\Users\lschlender\AppData\Local\Microsoft\Windows\Temporary%20Internet%20Files\Content.Outlook\4XGBQ7N7\Metformin%20diabetes%20older%20adults%20systematic%20review_clean_ARG.docx#_ENREF_38)] | Adjusted odds ratios | Renal function, and body mass index, duration of diabetes, duration of heart failure, and whether the patient developed diabetes or heart failure first, age, systolic blood pressure, diastolic blood pressure, body mass index,  hemoglobin A1c, GFR, hypertension, prior myocardial infarct or coronary revascularization, valvular heart disease, angina, atrial fibrillation, dyslipidemia, chronic obstructive lung disease, cerebrovascular disease, smoker (current or former), peripheral vascular disease, cancer,  dementia, peptic ulcer disease, medication use within 90 days of index date: angiotensin-converting-enzyme inhibitor/angiotensin receptor blockers, aspirin, digoxin, ß-blocker, statin, spironolactone |
| Masoudi 2005 [[39](file:///C:\Users\lschlender\AppData\Local\Microsoft\Windows\Temporary%20Internet%20Files\Content.Outlook\4XGBQ7N7\Metformin%20diabetes%20older%20adults%20systematic%20review_clean_ARG.docx#_ENREF_39)] | Adjusted hazards ratios | Demographic characteristics, medical history, clinical condition on presentation, laboratory evaluations, medications at discharge, characteristics of the treating attending physician, and hospital, admission blood glucose level and diabetes complications (ascertained with ICD-9 codes in discharge abstracts for the index hospitalization or in any part a record during the prior year), nephropathy, retinopathy, neuropathy, peripheral circulatory disorders, sulfonylureas and insulin, heart failure medication and associated conditions, including angiotensin-converting-enzyme inhibitors, adrenergic receptor blockers, statins |
| Moore 2013 [[40](file:///C:\Users\lschlender\AppData\Local\Microsoft\Windows\Temporary%20Internet%20Files\Content.Outlook\4XGBQ7N7\Metformin%20diabetes%20older%20adults%20systematic%20review_clean_ARG.docx#_ENREF_40)] | Adjusted odds ratios | Age, sex, level of education, and depression |
| Roumie 2012 [[41](file:///C:\Users\lschlender\AppData\Local\Microsoft\Windows\Temporary%20Internet%20Files\Content.Outlook\4XGBQ7N7\Metformin%20diabetes%20older%20adults%20systematic%20review_clean_ARG.docx#_ENREF_41)] | Adjusted hazards ratios | Age, sex, race, fiscal year of cohort entry, physiologic variables closest to cohort entry (blood pressure, creatinine, HbA1c, low density lipoprotein, body mass index, indicators of healthcare utilization (number of outpatient visits and active medications, hospitalization during baseline [yes/no]), smoking, selected medications indicative of cardiovascular disease and presence of comorbidities (myocardial infarction, obstructive coronary disease or prescription for a long acting nitrate, stroke/ transient ischemic attack, atrial fibrillation/ flutter, mitral/ aortic or rheumatic heart disease, asthma/obstructive pulmonary disease, procedures for carotid/ peripheral artery revascularization or bypass or lower extremity amputation, facility of care |
| Roussel 2010 [[42](file:///C:\Users\lschlender\AppData\Local\Microsoft\Windows\Temporary%20Internet%20Files\Content.Outlook\4XGBQ7N7\Metformin%20diabetes%20older%20adults%20systematic%20review_clean_ARG.docx#_ENREF_42)] | Adjusted hazards ratioss | Age and sex, propensity, geographic region, ethnic origin, education, employment, hypercholesterolemia, carotid surgery, atrial fibrillation or flutter, congestive heart failure, aortic valve stenosis, abdominal aortic aneurysm, body mass index, systolic blood pressure, and use of antiplatelet agents, anticoagulants, lipid-lowering agents, other cardiovascular agents |
| Schweizer 2009 [[43](file:///C:\Users\lschlender\AppData\Local\Microsoft\Windows\Temporary%20Internet%20Files\Content.Outlook\4XGBQ7N7\Metformin%20diabetes%20older%20adults%20systematic%20review_clean_ARG.docx#_ENREF_43)] | Adjusted mean differences | Hemoglobin A1c at baseline |
| Solomon 2009 [[44](file:///C:\Users\lschlender\AppData\Local\Microsoft\Windows\Temporary%20Internet%20Files\Content.Outlook\4XGBQ7N7\Metformin%20diabetes%20older%20adults%20systematic%20review_clean_ARG.docx#_ENREF_44)] | Adjusted relative risk | Age, gender, and race, diabetes-related, osteoporosis-related, and health care utilization variables, diagnoses of peripheral neuropathy, end-stage renal disease, retinopathy, and diabetic gastroparesis, diabetes-related hospitalizations, and number of visits for diabetes, a history of a fracture, the diagnosis of osteoporosis, rheumatoid arthritis, or hyperparathyroidism, an emergency room visit for a fall, and use of oral steroids or agents for osteoporosis (alendronate, calcitonin, etidronate, ibandronate, estrogen replacement therapy, raloxifene, risedronate, and teriparatide), congestive heart failure hospitalizations because of thiazolidinedione contraindication, year of the index date |
| Tzoulaki 2009 [[45](file:///C:\Users\lschlender\AppData\Local\Microsoft\Windows\Temporary%20Internet%20Files\Content.Outlook\4XGBQ7N7\Metformin%20diabetes%20older%20adults%20systematic%20review_clean_ARG.docx#_ENREF_45)] | Adjusted hazards ratios (aHR) | Sex and duration of diabetes, previous complications from diabetes, previous peripheral artery disease, previous cardiovascular disease, and coprescribed drugs, body mass index, cholesterol concentration, systolic blood pressure, HbA1c, creatinine concentration, albumin concentration, and smoking status. |

1. adjusted odds ratios (table 2) = 3, adjusted hazards ratios (aHR) = 7, Adjusted mean differences = 2, adjusted relative risk = 1, non reported = 2 [↑](#endnote-ref-1)
